# A new species and first record of the genus *Triacanthella* Schäffer, 1897 (Collembola, Poduromorpha, Hypogastruridae) for Africa

**DOI:** 10.3897/zookeys.163.2298

**Published:** 2012-01-09

**Authors:** Charlene Janion, Cyrille A. D'Haese, Louis Deharveng

**Affiliations:** 1 Centre for Invasion Biology, Department of Botany and Zoology, Stellenbosch University, Private Bag x1, Matieland, 7602, South Africa; 2 UMR7205 CNRS, Département Systématique et Évolution, Muséum National d’Histoire Naturelle, CP50, 45 rue Buffon, Paris 75005, France

**Keywords:** South Africa, Western Cape, gondwanian relict, cave, guano

## Abstract

The first species of the genus *Triacanthella* to be recorded from Africa is described. *Triacanthella madiba* **sp. n.** belongs to the Southern Hemisphere group of the genus. It is morphologically closely related to *Triacanthella vogeli* Weiner & Najt, 1997 from Chile, and appears to be a gondwanian relict. The new species is also the first *Triacanthella* recorded from a guano habitat.

## Introduction

The Cape Floristic Region in Western Cape Province of South Africa is the smallest Floral Kingdom in the world. Although its extraordinary rich flora has been well documented (Rebelo et al. 2006), studies have shown that its arthropods are not well known (Pryke and Samways 2008). Recent and extensive sampling of the Collembolan fauna of this region, undertaken within a bilateral project between South Africa and France, provided a wealth of new taxa in all groups (Janion et al. 2011). One of the most interesting discoveries was that of a new species of *Triacanthella*, a genus unrecorded from Africa so far. Here we provide the description of this new South African species along with its biogeographical considerations.

The genus *Triacanthella* is phyletically isolated among Hypogastruridae both from a morphological and a molecular point of view (D’Haese 2002, 2003a, 2003b; Greenslade et al. 2011). It contains 22 species with an intriguing distribution, reflecting a complex and probably ancient history: six including the generotype are found in Europe and Asia, eight species in Australia and New-Zealand, seven species in South America and one species in North America. Almost all species are endemic or micro-endemic and restricted to narrow ecological niches (Salmon 1941; de Izarra 1971; Cassagnau and Deharveng 1974). They mostly occur in epigean habitats, from warm littoral habitats to permanently cold habitats of high Mediterranean mountains and humid and fresh lowland habitats in southern Australia and New-Zealand. The species *Triacanthella perfecta* Denis, 1926 has been found once in a cave in southern France, but its normal habitat is forest litter (Arbea and Jordana 1991). Christiansen and Bellinger (1980) also recorded *Triacanthella copelandi* (Wray, 1963) once from a cave, but its type locality is not given as a cave in the original description, and there has been no other records of the species so far. The presence of a *Triacanthella* species in the guano of an African cave is therefore a surprising and important discovery.

## Materials and methods

The terminology used in the text follows D’Haese (2003a, b), and Fjellberg (1984, 1999) for mouthparts. Abbreviations used in description – **AIIIO**, organite of Ant. III; **Abd. I-VI**, abdominal segments, **Ant. I-IV**, antennal segments; **ms**, S-microchaeta; **S,** S-chaetae; **Th. I-III**, thoracic segments; **Md**, dorsal macrochaeta; **Mdl,** dorso-lateral macrochaeta; **hr**, anal valve chaetae.

### Identification key to the Southern Hemisphere *Triacanthella* species

**Table:** 

0	Sixth abdominal tergum with rosette-shaped tubercles, South America only	1
–	Sixth abdominal tergum without rosette-shaped tubercles	5
1	Empodium absent, claw without inner tooth	2
–	Empodium present (but rudimentary), claw with two inner teeth	3
2	Ommatidia G similar in size to the other ommatidia, Argentina	*Triacanthella michaelseni* Schäffer, 1897
–	Ommatidia H and G reduced compared to the other ommatidia, Argentina	*Triacanthella rosae* Wahlgren, 1906
3	Posterior anal spine less than half the size of the other two, dentes without apical lobe, Chile	*Triacanthella vogeli* Weiner & Najt, 1997
–	Posterior anal spine at least half the size of the other two, dentes with distinct apical lobe	4
4	Macrochaetae long, half tergite macrochaetal chaetotaxy = 7 / 2, 3, 3 / 4, 4, 4, 4, Chile and Argentina	*Triacanthella andina* Cassagnau & Rapoport, 1962
–	Macrochaetae short, half tergite macrochaetal chaetotaxy = 8 / 2, 4, 4 / 3, 3, 3, 3, Argentina	*Triacanthella najtae* de Izarra, 1971
5	Posterior anal spine at least half the size of the other two	6
–	Posterior anal spine less than half the size of the other two	12
6	Mucro reduced to a small projection (i.e. almost absent)	7
–	Mucro well developed	8
7	Colour in alcohol pinkish, Campbell Island	*Triacanthella sorenseni* Salmon, 1949
–	Colour in alcohol whitish-yellowish, Campbell Island	*Triacanthella alba* Carpenter, 1906
8	Mucro simple and straight	9
–	Mucro more complex with two teeth	10
9	All ommatidia equally developed, tibiotarsi with clavate tenent hair, Argentina	*Triacanthella massoudi* Najt, 1973
–	Two ommatidia (G and H) absent, tibiotarsi without clavate tenent hair, Australia	*Triacanthella violacea* Womersley, 1939
10	Macrochaetae simple and smooth, New Zealand	*Triacanthella rubra* Salmon, 1941
–	Macrochaetae serrated or brush-like	11
11	All ommatidia equally developed, apical lobe absent on dentes, New Zealand	*Triacanthella purpurea* Salmon, 1943
–	Two ommatidia (G and H) reduced, apical lobe present on dentes, New Zealand	*Triacanthella enderbyensis* Salmon, 1949
12	Two ommatidia (G and H) reduced	13
–	All ommatidia equally developed	14
13	Dentes reduced, empodium present (but rudimentary), Chile	*Triacanthella clavata* (Willem, 1902)
–	Dentes normally developed, empodium absent, New Zealand	*Triacanthella terrasilvatica* Salmon, 1943
14	Mucro more complex with two teeth, colour whitish-yellowish in alcohol, claw without inner tooth, New Zealand	*Triacanthella setacea* Salmon, 1941
–	Mucro with a distinct heel, colour pinkish in alcohol, claw with two inner teeth, South Africa	*Triacanthella madiba* sp. n.

## Species description

### Triacanthella madiba sp. n.

urn:lsid:zoobank.org:act:606016FB-A5C4-4B86-A9EC-E111EB7CCAEB

http://species-id.net/wiki/Triacanthella_madiba

#### Material.

Holotype female and 17 paratypes (9 on slides and 8 in alcohol), South Africa: Western Cape, Cape Town, Table Mountain National Park, 10 March 2009, bat guano in Wynberg cave, extracted on Berlese-Tullgren funnel, (SAF-125, Louis Deharveng & Anne Bedos leg).

Holotype on slide and 9 paratypes (5 on slides and 4 in alcohol) in Iziko Museum (Cape Town, South Africa), 8 paratypes in Museum National d’Histoire Naturelle, Paris (4 on slides and 4 in alcohol).

#### Description.

**Figure 1.**
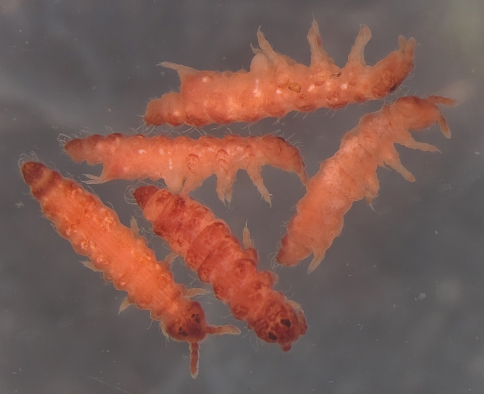
*Triacanthella madiba* sp. n., aspect and colour after one year in 95% ethanol.

Colour orange to pink alive, pinkish in ethanol even after one year (Fig. 1). Length 1.9 – 2.5 mm. Habitus of Southern Hemisphere *Triacanthella* (Figs 1, 6A).

**Figure 2.**
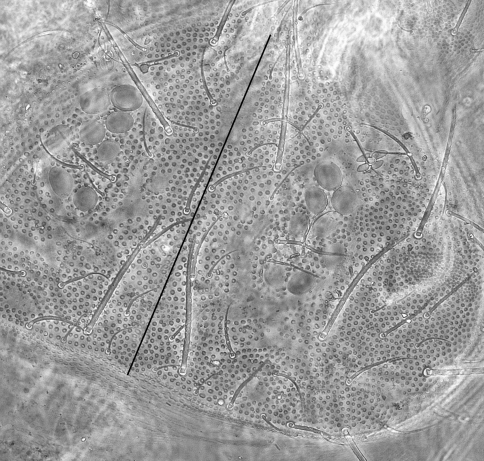
*Triacanthella madiba* sp. n., dorsal side of head.

**Figure 3.**
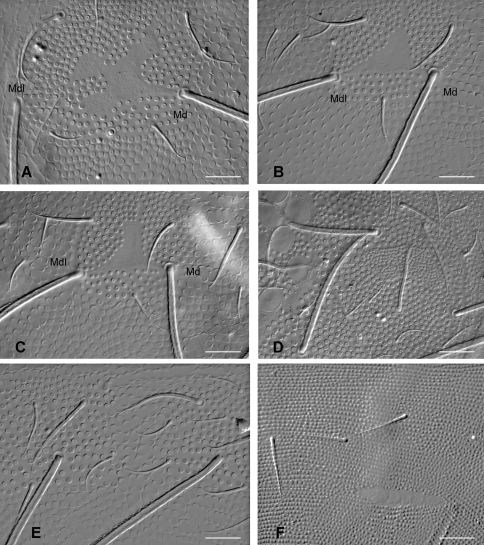
*Triacanthella madiba* sp. n., details of granulation types on dorsal side of the body. **A** amiboid primary granule area on Th. III **B** triangular primary granule area on Abd. III **C** triangular primary granule area on Abd. II, surrounded by smaller secondary granules **D** detail of the lateral plate of smaller secondary granules on head **E** axial area of Abd. V, with larger secondary granules between axial chaetae **F** linear arrangement of secondary granules on the manubrium. Scales: 30 µm.

**Figure 4.**
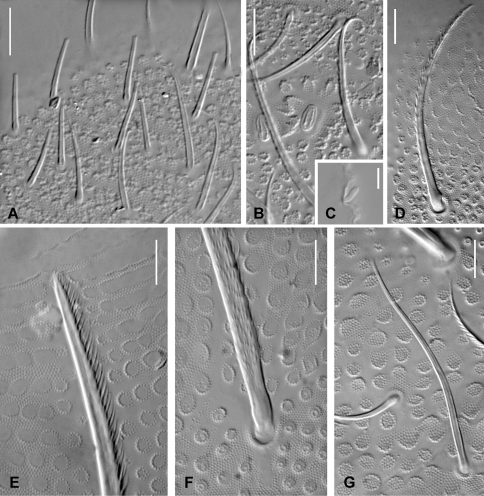
*Triacanthella madiba* sp. n., types of chaetae. **A** truncated chaetae of the ventral side of Ant. IV **B** microchaetae S3 and S4 of AIIIO **C** S-microchaeta of Ant. IV **D** ciliated chaeta of Ant. III **E** distal part of a macrochaeta on Abd. I **F** basal part of a macrochaeta on Abd. III **G** S-chaeta on Abd. I. Scales: 10 µm (**A, B, D, E, F, G**); 5 µm (**C**).

Dorsal integument ornamentation made of hemispherical and rather coarse secondary granules, with large areas devoid of secondary granules on head and tergites (Figs 2–3), symmetrically arranged; most noticeable are the long antero-axial one on head, those associated to classical suture zone of head (Fig. 2), the 1+1 amiboid ones on Th. II-III (Fig. 3A), and the triangular ones between Md and Mdl on Abd. I-III (Figs 3B-C); secondary granules smaller around these areas. Externally to ocular area is a large area where secondary granules are smaller and denser (Fig. 3D). Secondary granules larger along the axial zone (Fig. 3E). No rosette-like arrangement of secondary granules on Abd. VI. Ventral secondary granulation less coarse, more regular. Manubrium with secondary granules arranged in a characteristic linear pattern dorsally (Fig. 3F), and with large areas devoid of secondary granulation ventrally. Pseudopores not seen. Chaetotaxy characterized by a strong heterochaetosis dorsally and a moderate plurichaetosis on most body parts. Chaeta morphology described below, with macrochaetae, mesochaetae and S-chaetae on head and body, and various kinds of chaetae on antennae (Figs 4, 6C). No ordinary microchaetae except on praetarsus and genital plate.

Antennae almost as long as head diagonal. Six kinds of antennal chaetae: i) thickened subcylindrical S-chaetae of medium size (2 on Ant. III and 6 on Ant. IV); (ii) S-microchaetae (3 on Ant. III and 1 on Ant. IV) (Figs 4B–C); (iii) blunt chaetae very similar to the S-chaetae, but longer and usually slightly thinner (on Ant. IV); (iv) acuminate ordinary chaetae of various length, smooth or weakly serrated, 11–12, 13–17 and 26–30 on Ant I-III, a few on Ant. IV (Fig. 4D); (v) thin, straight and smooth truncated chaetae numerous ventrally on Ant. IV (Fig. 4A); (vi) one ventro-distal papillate chaeta. Sensory organ of AIIIO with two short S-chaetae lying in ovoid sockets (S3 and S4, Fig. 4B), two longer guard S-chaetae shorter than nearest mesochaetae (S2 and S5) and one very small dorso-external S-microchaeta (S1); integument granulation significantly coarser between and above S3 and S4 (Fig. 4B). Antennal segment IV with most chaetae as subcylindrical, thickened, blunt S-chaetae, the shortest ones slightly thicker and more bent, including a central group of six; apical bulb trilobed; subapical organite rounded, very small; a short ovoid-elongate S-microchaeta present dorso-externally (Figs 4C, 6B).

**Figure 5.**
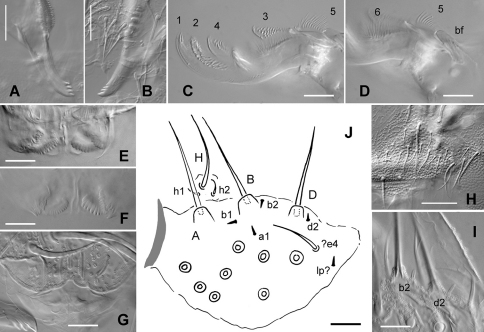
*Triacanthella madiba* sp. n., mouthparts. **A** mandible, right **B** mandible, left **C** maxilla head **D** proximal part of maxilla head, with basal flap bf **E, F** ventro-distal part of labrum with combs **G** dorso-distal part of labrum **H** clypeus **I** guards b2 and d2 of labial palp **J** labial palp (lp: lateral process). Scales: 10 µm.

Eight ocelli on each side of the head, equal in size. Postantennal organ nearly equal in size to one ocellus, with 4 subequal vesicles (Fig. 2). Maxilla with a tridentate capitulum, a rounded basal flap and 6 variously fringed or ciliate lamellae (Figs 5C–D). Mandible head with 4 teeth on each side, the basal one slightly smaller on the left than on the right mandible (Figs 5A–B). Labrum chaetotaxy 4/4,5,4; labral chaetae distinctly longer than prelabral chaetae; labrum apical edge with a slight medial indentation; distal part with four irregular longitudinal ridges dorsally, and with subapical asymmetrical combs ventrally (Figs 5E–G); labral apical edge hemmed (Fig. 5G). Labium with 5–6 basomedian chaetae, 7 lateral chaetae, and a labial palp characterized by 7–8 proximal chaetae and a reduced number of distal chaetae (Fig. 5J): only 3 papillae, A,B,D; one ordinary chaeta (possibly e4, but with a socket) and 5 short, thickened, hyaline guards (a1, b1, b2, d2, Fig. 5I), with the fifth one probably the lateral process sensu Fjellberg (1999). Maxillary outer lobe with one basal chaeta and a simple palp; sublobal plate small, rounded and devoid of sublobal hairs. Clypeus with 15–16 chaetae (Fig. 5H). Postlabial chaetae 5+5.

**Figure 6.**
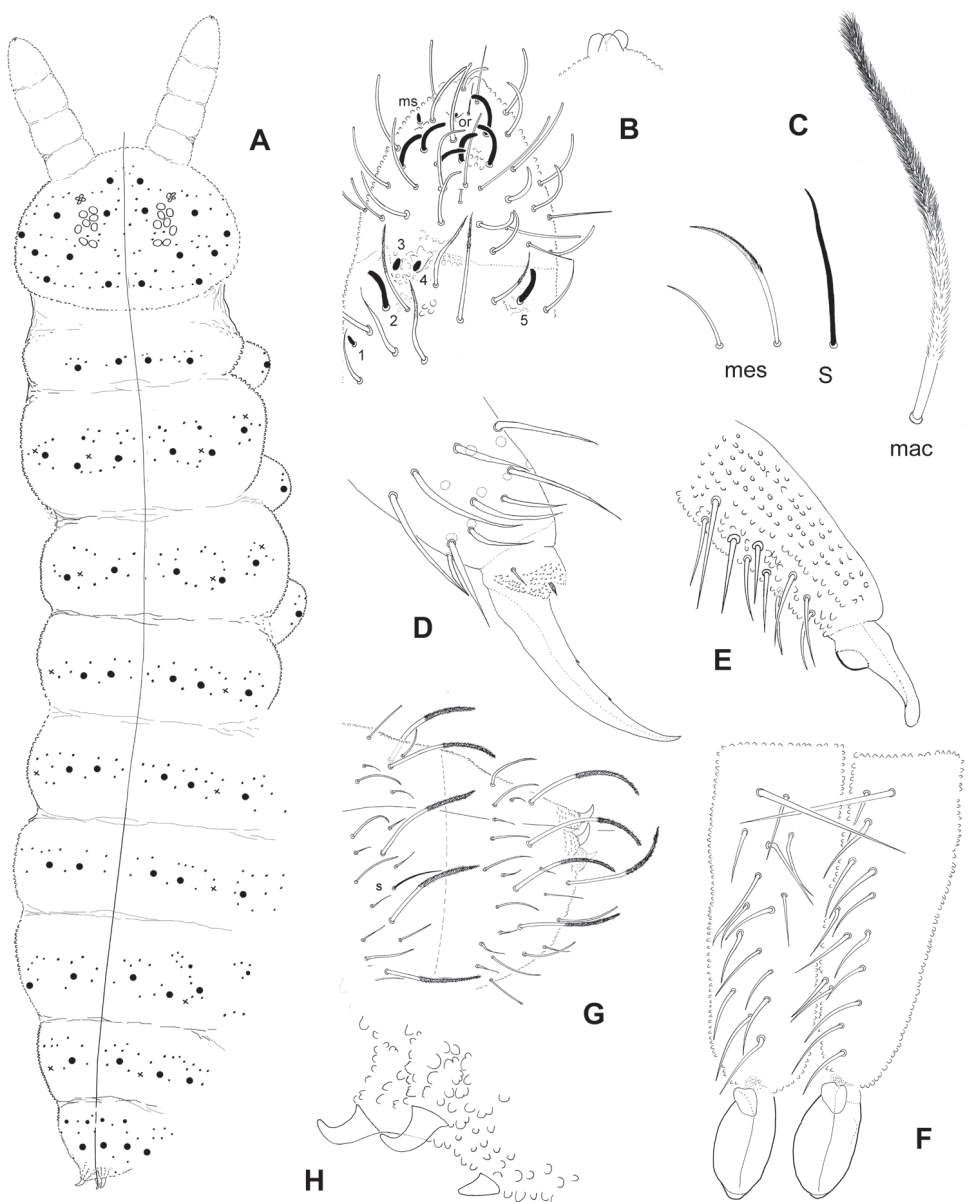
*Triacanthella madiba* sp. n. **A** habitus and chaetae distribution of the dorsal side (x: S-chaetae) **B** Ant. III distal and Ant. IV in dorsal view and detail of the apical bulb **C** morphology of dorsal chaetae: macrochaeta (mac), mesochaeta (mes), S-chaeta (S) **D** tibiotarsus and claw of leg III **E** mucrodens, lateral view **F** Mucrodens, dorsal view **G** Abd. V–VI tergites **H** anal spines.

Chaetotaxy of tergites illustrated on Fig. 6A. Dorsal clothing plurichaetotic and heterochaetotic, with frequent asymmetries among shortest mesochaetae. Macrochaetae long, bent, and densely ciliated unilaterally on 2/3 to most of their length; mesochaetae, less bent, acuminate, less strongly ciliated to almost smooth; S-chaetae, thin and smooth, shorter than macrochaetae (Figs 4E–G, 6C). Macrochaetae formula per half-tergite: 8/2,3,3/3,3,3,3(4),3. Number of chaetae between macrochaetae Md per half-tergite: 1-2,3-5,3-4/2-3,2-3,(1)-2,3,2-3 with many asymmetries. S-chaetae formula per half-tergite: 0,2,2/1,1,1,1,1; microchaeta ms absent. Abdomen VI chaetotaxy often asymmetrical, with one or two axial short mesochaetae; three anal spines on papillae, the posterior one less than half the length of the other two which are hook-like (Figs 6G–H).

No ventral chaetae on thoracic sternites. Number of ventral chaetae per half-tergite for Abd. II, III: 7, 13–17; anterior furcal subcoxa with 12–16 chaetae. All ventral chaetae are smooth ordinary chaetae. Lateral anal valves with 3 or 4 hr chaetae; upper anal valve with 7–9 hr chaetae.

Leg chaetotaxy slightly plurichaetotic. Trochanter with 7 chaetae. Tibiotarsi I, II, III with (proximal + distal): 8 + 11, 8 + 11, 7 + 11 acuminate chaetae. No clavate tenent hair. Claw with two inner teeth at about 40% and 65% of claw basis, and 1 + 1 latero-distal teeth, appressed on the integument and difficult to see at about 85% of claw basis (Fig. 6D). Empodial appendage short and pointed, internal to empodial apical tubercle according to Fig. 6D, 1+1 small praetarsal microchaetae. Ventral tube with 9–11 + 9–11 latero-distal chaetae, and 1–2 chaetae on each side of the sternite of Abd. I. Tenaculum with 3 + 3 teeth. Dens without ventro-apical lobe, bearing 10–15 chaetae dorsally with fine granulation (secondary granules smaller than chaetal sockets); the basal macrochaeta of the dens is about 2.3 the length of the nearest mesochaeta; well developed mucro with a large lamella and a very distinct dorso-basal heel (Figs 6E–F).

#### Discussion.

*Triacanthella madiba* sp. n. shares numerous characters with *Triacanthella vogeli* Weiner & Najt, 1997, described from southern Chile. It differs mainly by the ocelli G and H being equal in size to the other ocelli and the absence of rosette-shape tubercles on Abd. VI. It is also morphologically close to *Triacanthella andina* Cassagnau & Rapoport, 1962 from Argentina, but macrochaetae are less numerous on Abd. I-III "(333 versus 444). In addition, the lamellae of the maxilla are shorter and the papillae bearing the anal spines are not as strong in *Triacanthella madiba* sp. n., as in *Triacanthella andina*. Overall, these three species are extremely similar morphologically in spite of being very remote geographically. *Triacanthella madiba* sp. n. differs from Australian and New-Zealand species by characters pointed out in the key. An additional important character is the chaetotaxy of the distal part of the labial palp, which is similar to that described by Fjellberg for an unidentified species of Australia (Fjellberg 1999), being strongly reduced compared to that of *Triacanthella biroi*, Stach 1924 from Europe (Fjellberg 1999).

#### Distribution and ecology.

*Triacanthella madiba* sp. n. is recorded in bat guano in a cave of Table Mountain National Park. This is the first record of the genus *Triacanthella* in a guano habitat and the first record of the genus for Africa. None of the *Triacanthella* species recorded so far are found in tropical regions. They are all restricted to temperate zones, where they occur in a large range of habitats in Europe (from xeric Mediterranean to permanently cold), while they are limited to humid and cool litter or surface soil layers in the southern hemisphere (Australia, New Zealand, Chile and Argentina, Weiner and Najt 1997). Its presence underground in a cool, nutrient rich and permanently humid habitat, and its absence in the remnant forest patches of Table Mountain that we sampled extensively is surprising.

The only subterranean records of the genus *Triacanthella*, include the record of *Triacanthella copelandi* in a cave in Tennessee (USA), without anymore detail, and a single specimen collected in a small shallow cave of oriental Pyrenees in France, that was described as *Triacanthella proxima* Delamare 1951, and later synonymised with *Triacanthella perfecta*. In the area around this last cave, *Triacanthella perfecta* is actually common in beech forest litter (unpublished observations), and its presence underground as a single specimen is obviously accidental. Conversely, *Triacanthella madiba* sp. n. occurs abundantly in the guano microhabitat of Wynberg cave and was not found outside in Table Mountain. The species can therefore be considered troglophilic in this area. Actually, *Triacanthella madiba* sp. n. may have been already recorded as *Schaefferia* (*Typhlogastrura*) sp. in Sharratt et al. (2000), collected from guano material during a cave fauna survey in Table Mountain.

Although the labial palp of *Triacanthella madiba* sp. n. is similar to the unidentified Australian species (Fjellberg 1999), it also shares some characters with certain South American species, making its placement in the phylogeny based on morphological characters problematic. If the new species is more closely related to the Australian and New-Zealand species, it would follow a classical transantarctic gondwanian pattern (Brundin 1965, 1966; Sanmartín and Ronquist 2004). Alternatively, it could be that *Triacanthella madiba* sp. n. is more closely related to South American species based on the characters pointed out in the key provided. Thus, *Triacanthella madiba* sp. n. could be the result of dispersal from South America (e.g. Allwood et al. 2010), associated with a significant shift in its ecological requirements. On-going molecular and morphological studies on this basal genus will hopefully resolve their intriguing biogeographic pattern.

#### Name derivation.

We dedicate this species to Madiba, former President of South Africa, Nelson Rolihlahla Mandela, who celebrated his 20 years of freedom on 11 February 2010.

## Supplementary Material

XML Treatment for Triacanthella madiba

## References

[B1] Arbea JI Jordana R (1991) Colémbolos de Navarra (norte de la Península Ibérica). I. Orden Poduromorpha (Collembola). Publicacíones de Biología de la Universidad de Navarra, Serie Zoologica 22: 1-149.

[B2] Allwood J Gleeson D Mayer G Daniels S Beggs JR Buckley TR (2010) Support for vicariant origins of the New Zealand Onychophora. Journal of Biogeography 37: 669-681. doi: 10.1111/j.1365-2699.2009.02233.x

[B3] Brundin L (1965) On the real nature of transantarctic relationships. Evolution 19: 496-505. doi: 10.2307/2406246

[B4] Brundin L (1966) Transantarctic relationships and their significance, as evidenced by chirono- mid midges, with a monograph of the subfamilies Podonominae and Aphroteniinae and the austral Heptagyiae. Kungliga Svenska Vetenskapsakademiens Handlingar 11: 1-472.

[B5] Cassagnau P Deharveng L (1974) Les espèces européennes du genre *Triacanthella* (Collemboles). Nouvelle Revue d'Entomologie 4: 165-180.

[B6] Christiansen K Bellinger P (1980) Part I. Poduridae and Hypogastruridae, The Collembola of North America North of Rio Grande, Grinnell College, Iowa, 386 pp.

[B7] de Izarra DC (1971) Sobre el genero *Triacanthella* Schäffer con descripcion de una nueva especie: *T. najtae* (Insecta, Collembola). Physis 30: 345-350.

[B8] D’Haese CA (2002) Were the first springtails semi-aquatic? A phylogenetic approach by means of 28S rDNA and optimization alignment. Proceedings of the Royal Society of London B 269: 1143-1151. doi: 10.1098/rspb.2002.1981 PMC1691003 12061958

[B9] D’Haese CA (2003a) Homology and morphology in Poduromorpha (Hexapoda, Collembola). European Journal of Entomology 101: 385-407.

[B10] D’Haese CA (2003b) Morphological appraisal of Collembola phylogeny with special emphasis on Poduromorpha and a test of the aquatic origin hypothesis. Zoologica Scripta 32: 563-586. doi: 10.1046/j.1463-6409.2003.00134.x

[B11] Fjellberg A (1984) Maxillary structures in Hypogastruridae (Collembola). Annales de la Société Royale de Zoologie de Belgique 114: 89-99.

[B12] Fjellberg A (1999) The labial palp in Collembola. Zoologischer Anzeiger 237: 309-330.

[B13] Greenslade P Stevens MI Torricelli G D’Haese CA (2011) An ancient Antarctic endemic genus restored: morphological and molecular support for *Gomphiocephalus hodgsoni* (Collembola: Hypogastruridae). Systematic Entomology 36: 223-240. doi: 10.1111/j.1365-3113.2010.00553.x

[B14] Janion C Bedos A Bengtsson J Deharveng L Jansen van Vuuren B Leinaas HP Liu A Malmström A Porco D Chown SL (2011) Springtails diversity in South Africa. South African Journal of Science 107 (11/12): 1-7. doi: 10.4102/sajs.v107i11/12.582

[B15] Miller JS Kamath A Damashek J Levin RA (2011) Out of America to Africa or Asia: Inference of dispersal histories using nuclear and plastid DNA and the S-RNase self-incompatibility locus. Molecular Biology and Evolution 28: 793-801. doi: 10.1093/molbev/msq253 20855430

[B16] Pryke JS Samways MJ (2008) Conservation of invertebrate biodiversity on a mountain in a global biodiversity hotspot, Cape Floral Region. Biodiversity and Conservation 17: 3027-3043. doi: 10.1007/s10531-008-9414-4

[B17] Rebelo AG Boucher C Helme N Mucina L Rutherford MC (2006) Fynbos Biome. In: Mucina L Rutherford MC (Eds). The Vegetation of South Africa, Lesotho and Swaziland. South African National Biodiversity Institute, Pretoria: 52-219.

[B18] Salmon JT (1941) The Collembolan Fauna of New Zealand, including a discussion of its distribution and affinities. Transactions of the Royal Society of New Zealand 70: 282-431.

[B19] Sharratt NJ Picker MD Samways MJ (2000) The invertebrate fauna of the sandstone caves of the Cape Peninsula (South Africa): patterns of endemism and conservation priorities. Biodiversity and Conservation 9: 107-143. doi: 10.1023/A:1008968518058

[B20] Weiner WM Najt J (1997) Collembola Poduromorpha from the Magallanes Province (Chile). Bonner Zoologische Beiträge 47: 99-110.

